# Expanding the clinical and genetic spectrum of Heimler syndrome

**DOI:** 10.1186/s13023-019-1243-x

**Published:** 2019-12-12

**Authors:** Feng-Juan Gao, Fang-Yuan Hu, Ping Xu, Yu-He Qi, Jian-Kang Li, Yong-Jin Zhang, Fang Chen, Qing Chang, Fang Song, Si-Mai Shen, Ge-Zhi Xu, Ji-Hong Wu

**Affiliations:** 1 Eye Institute, Eye and ENT Hospital, College of Medicine, Fudan University, Shanghai, China; 2 Shanghai Key Laboratory of Visual Impairment and Restoration, Science and Technology Commission of Shanghai Municipality, Shanghai, China; 3 Key Laboratory of Myopia (Fudan University), Chinese Academy of Medical Sciences, National Health Commission, Shanghai, China; 4 BGI-Shenzhen, Shenzhen, China; 5 Department of Computer Science, City University of Hong Kong, 83 Tat Chee Ave, Kowloon, Hong Kong; 6 Laboratory of Genomics and Molecular Biomedicine, Department of Biology, University of Copenhagen, Copenhagen, Denmark; 7 Shenzhen Engineering Laboratory for Birth Defects Screening, BGI-Shenzhen, Shenzhen, China; 8 Sichuan University, Chengdu, China

**Keywords:** Heimler syndrome, Next-generation sequencing, Genetic diagnosis, *PEX1*, *PEX6*, Genotype–phenotype

## Abstract

**Background:**

Heimler syndrome (HS) is a rare hereditary systemic disorder, partial clinically overlapping with Usher syndrome. So far, our knowledge of HS is very limited, many cases are misdiagnosed or may not even be diagnosed at all. This study aimed to analyze the clinical and genetic characteristics of HS, and to evaluate potential phenotype–genotype correlations.

**Results:**

Two HS cases caused by *PEX1* mutations were identified, and a novel likely pathogenic mutation, *PEX1* c.895_896insTATA, was found. The main ophthalmic finding of the two patients was consistent with retinitis pigmentosa accompanied by cystoid macular edema, but short axial length and hyperopia were also observed as two previously unreported ocular phenotypes. Analysis of the literature showed that of the 29 HS patients previously reported, 12 had *PEX6* mutations, 10 had *PEX1* mutations, two had *PEX26* mutations, and the remaining patients were not genetically tested. Three novel genotype–phenotype correlations were revealed from analysis of these patients. First, most genotypes of every HS patient include at least one missense variant; second, at least one mutation in *PEX1* or *PEX6* gene affects the AAA-ATPase region in every HS patient with retinal dystrophy, suggesting AAA-ATPase region is a hypermutable region in patients with a retinal dystrophy; third, there are no significant differences between *PEX1*-, *PEX6*-, and *PEX26*-associated phenotypes.

**Conclusion:**

Next-generation sequencing is important for the diagnosis of HS. This study expands the clinical and genetic spectrum of HS, and provides additional insights into genotype–phenotype correlations, which is vital for accurate clinical practice, genetic counseling, and pathogenesis studies.

## Background

Heimler syndrome (HS) was first reported in 1991 by A. Heimler as an inherited syndrome characterized by sensorineural hearing loss (SNHL), enamel hypoplasia, and nail abnormalities [1]. Biallelic mutations in the peroxisomal biogenesis factor 1 gene (*PEX1*; MIM*602136), peroxisomal biogenesis factor 6 gene (*PEX6*; MIM*601498), and peroxisomal biogenesis factor 26 gene (*PEX26*; MIM* 608666) are responsible for HS [2–4]. The proteins they encode function together to control peroxisomal matrix protein import, and mutations of these genes are implicated in peroxisome biogenesis disorders (MIM phenotypic series PS214100). These are characterized by a deficiency of essential peroxisomal functions or even a complete loss of functional peroxisomes, resulting in a wide range of phenotypes that vary in severity [5, 6]. Patients with the most serious phenotype present at birth only live a few weeks or months (Zellweger syndrome) [7], while some disorders generally present later in childhood, primarily with leukodystrophy, SNHL, retinal dystrophy, and developmental and cognitive delay. Others may have multiple organ dysfunction and psychomotor impairments including craniofacial dysmorphism, neurological abnormalities, sensory defects, and liver, kidney, and bone abnormalities [5]. Therefore, patients with HS represent the mildest phenotypic subgroup [8, 9].

Our current knowledge of HS is very limited, with only 29 reported patients worldwide; 26 of these have genetic sequence information [1–4, 9–14]. HS is also a systemic disease with a variety of other co-existing congenital abnormalities, and diagnostic criteria have not been proposed [2, 15]. The HS clinical phenotype varies, but includes acquired SNHL, amelogenesis imperfecta of the teeth, and retinal dystrophy, partially clinically overlapping with Usher syndrome that is characterized by congenital deafness, retinitis pigmentosa (RP), presence or absence of vestibular dysfunction, but no dental anomalies [4]. Although the number of reported cases is small, this does not reflect a low incidence of disease; rather many cases are misdiagnosed or may not even be diagnosed at all, because of the clinical evaluation alone is especially difficult and often not definitive [4]. Thus, an evidence-based, early, accurate, and fast diagnostic approach is in great need. Fortunately, next-generation sequencing (NGS) methods have been shown to be a powerful tool for the diagnosis of genetic or presumed genetic disorders [16, 17]. In this study, we accurately diagnosed two HS patients using NGS and presented their comprehensive ophthalmic examinations. We further reviewed the varied phenotypes and genotypes of all previously reported cases, and uncovered novel genotype–phenotype correlations.

## Methods

### Subjects and ethics statement

The current study was performed in accordance with the Code of Ethics of the World Medical Association (Declaration of Helsinki) for medical research involving human subjects, and was approved by the Ethics Committee of the Eye & ENT Hospital of Fudan University. Two HS patients and their relatives were recruited after obtaining informed consent.

### Clinical evaluation and sample collection

Both patients underwent a full ophthalmic examination, including best corrected Snellen visual acuity testing (BCVA), color vision (Ishihara color plate), slit lamp biomicroscopy, tonometry, dilated fundus examination, ultrasound biomicroscopy (UBM), A/B-ultrasound (MD-300 L; MEDA Co., Ltd., Tianjin, China), wide-field fundus imaging (Optos PLC, Dunfermline, United Kingdom), spectral domain optical coherence tomography (SD-OCT, Spectralis HRA + OCT, Heidelberg, Engineering Inc., Heidelberg, Germany), full-field electroretinography (ERG, according to the standards of the International Society for Clinical Electrophysiology of Vision; available at www.iscev.org), multifocal ERG (mfERG, LKC Utas E3000 LKC Technologies Inc., USA), and visual field (Humphrey Visual Field Analyzer, Carl Zeiss Inc., CA, USA). Family and medical history including subjective degree of vision loss, age of onset and other related clinical manifestations were recorded. Blood samples of all the patients and their family members were collected from the peripheral blood and stored at 4°Cbefore further analysis.

### Genetic analyses and confirmation of suspected pathogenic variants

DNA samples were extracted from whole blood using the FlexiGene DNA Kit (Qiagen, Venlo, The Netherlands) according to the manufacturer’s protocol. All participants in this study were subjected to analysis using this panel-based NGS. The capture panel contains exon-capture and untranslated regions (UTRs) of 762 genes that are involved in common inherited eye diseases (BGI-Shenzhen, Additional file 3: Table S1). Acquisition of probe sequences: obtain the exon sequence and its flank ±30 bp of 762 genes from hg38. Each reference sequence begins at one end of a reference sequence and selects the reference sequence of the predetermined length to obtain the probe sequence so that the last total probe can cover the reference sequence at least once, and the probe length of the panel is 90 nt, the total target area obtained is 2.3 M. On average, the mean coverage depth was more than 400X and the coverage of target region was around 99.9% by using BGISEQ-500. Subsequent points for sample quality control have also been added to the probe design process. After sequencing, data analysis was performed as reported previously [18, 19]. Previously reported variants were determined using ClinVar (https://www.ncbi.nlm.nih.gov/clinvar/), the Human Gene Mutation Database (HGMD Professional 2018.4, http://www.hgmd.cf.ac.uk/ac/index.php), and locus-specific databases. Variants were classified as pathogenic and likely pathogenic according to the American College of Medical Genetics (ACMG) and genomics guidelines for the more recent cases. Before confirmation by Sanger sequencing, the candidate variants were reviewed by clinical geneticists and ophthalmologists. Segregation analysis was performed within family members.

## Results

### Genetic analyses of the two patients

**Fig. 1:**
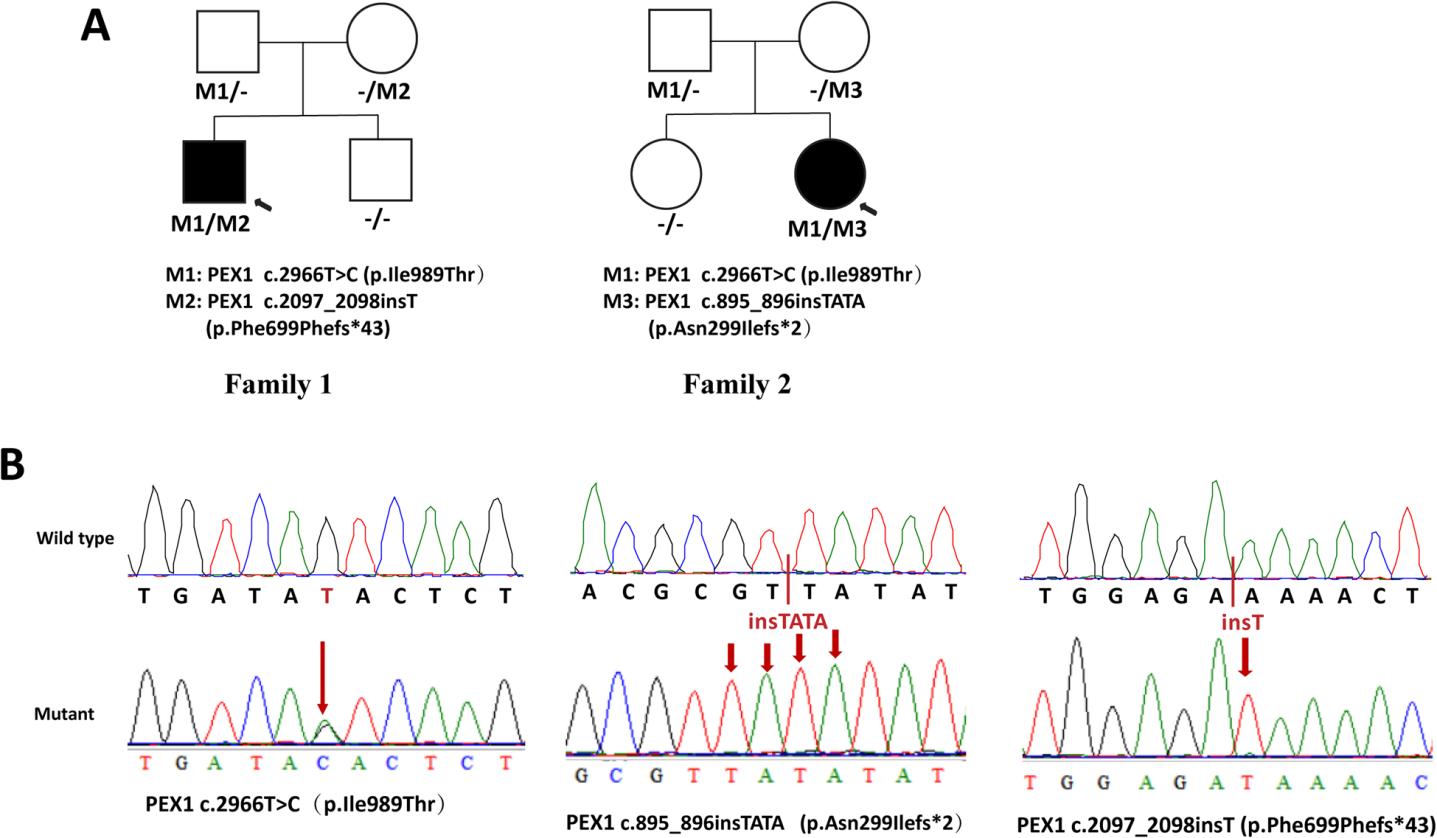
Pedigrees (**a**) and identified mutations (**a**). **a** Circles represent females, and squares represent males. Filled symbols represent affected patients, and empty symbols represent unaffected family members. **b** Sequencing results of the mutations in the *PEX*1 gene. Arrows indicate the position of the mutated nucleotide

Two previously reported mutations, c.2966 T > C (p.Ile989Thr, maternally inherited) and c.2097_2098insT (p.Phe699Phefs*43, paternally inherited) [2, 9], were found in patient 1. A known missense mutation, c.2966 T > C (p.Ile989Thr, maternally inherited) [2, 9], and a novel likely pathogenic mutation, c.895_896insTATA (p.Asn299Ilefs*2, paternally inherited), were found in patient 2. Pedigrees and identified mutations of the two families are shown in Fig. 1. We found no additional pathogenic or likely pathogenic variants known to be associated with inherited eye diseases in either patient.

### Clinical features of the two patients

**Fig. 2:**
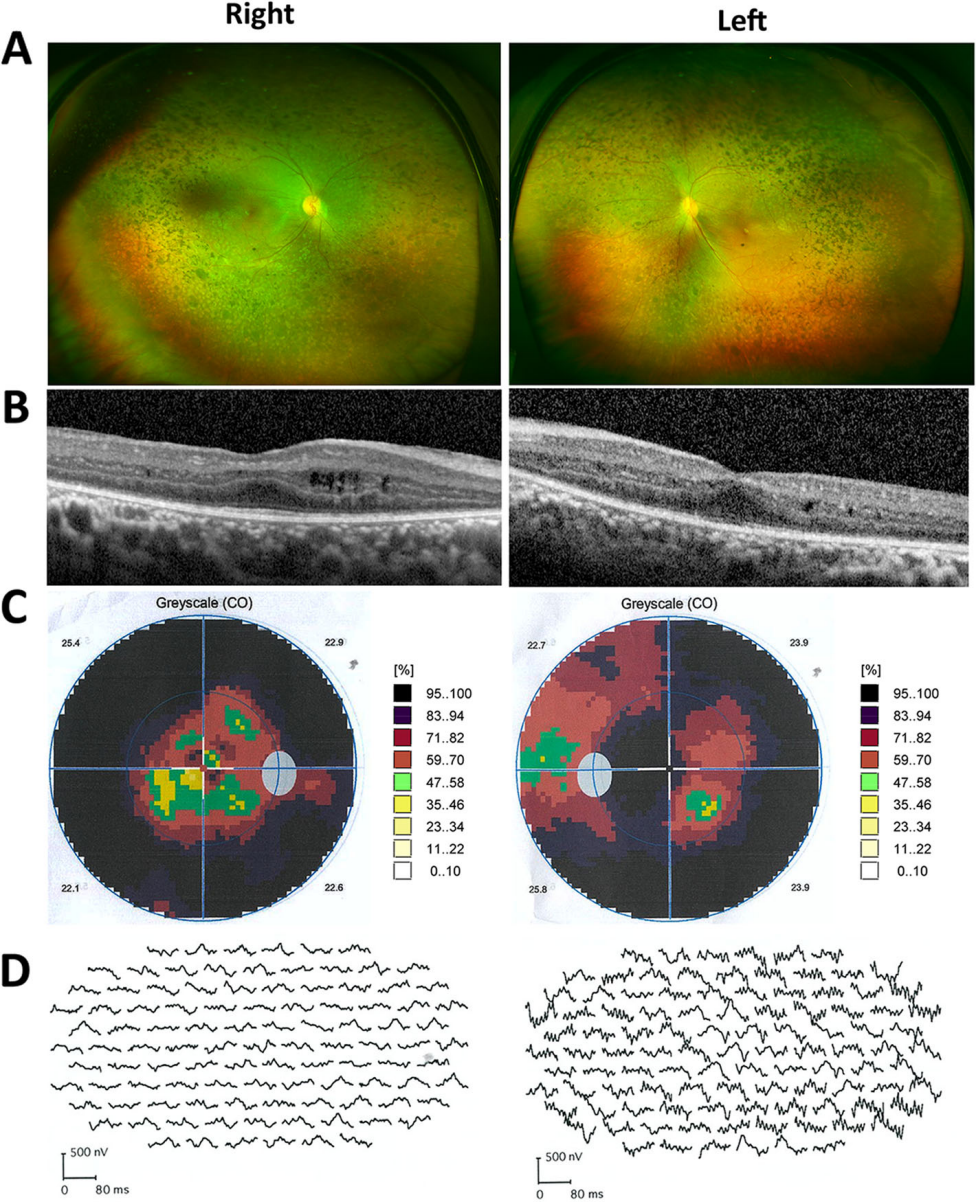
Ocular features of patient 1. **a** Ultra wide-field pseudocolor images showing retinal atrophy in the mid and far periphery combined with significant bone spicule-like pigmentation and mottling in both eyes. **b** SD-OCT shows small macular cysts in the inner nuclear layer, a thickened retina, and an atrophic photoreceptor layer with preservation of the ellipsoid zone (EZ) and the external limiting membrane (ELM). **c** Visual field shows peripheral visual field loss, with small central loss. **d** mfERG shows that amplitudes in the posterior-pole of both eyes were significantly reduced, more seriously in the right eye

**Fig. 3:**
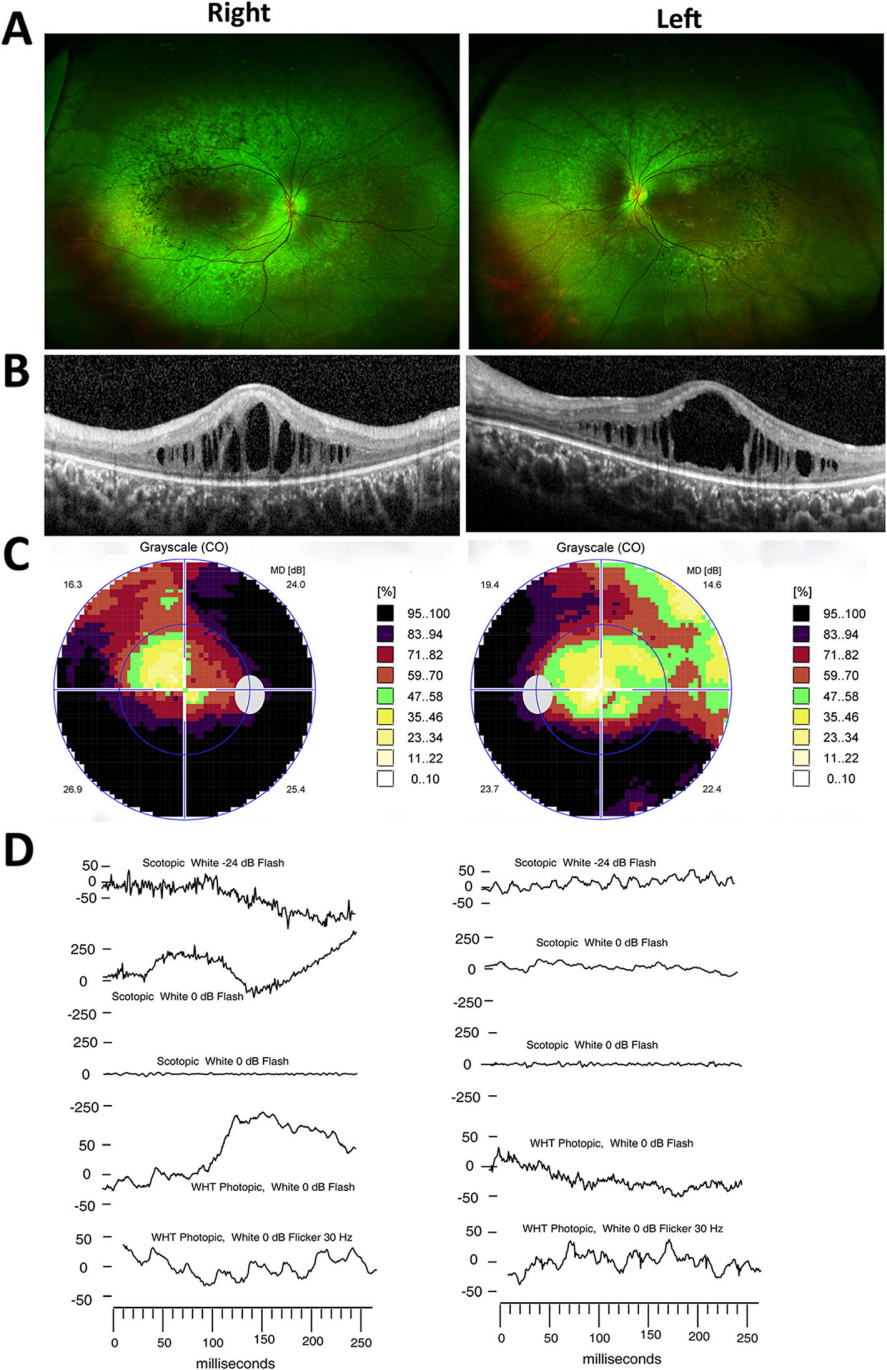
Ocular features of patient 2. **a** Ultra wide-field pseudocolor images showing retinal atrophy in the mid and far periphery combined with significant bone spicule-like pigmentation, mottling in both eyes. **b** SD-OCT shows bilateral cystoid macular edema, which mainly involved the outer nuclear layer (ONL). The foveal and peripheral macular ellipsoid zone (EZ) was disrupted and almost absent. **c** Visual field shows peripheral visual field loss except for the superior nasal quadrant. **d** mfERG shows undetectable rod ERG, subnormal bright flash ERG, and subnormal and delayed cone ERG

Patient 1 is a 9-year-old boy, and patient 2 is an 8-year-old girl. Clinical characteristics of the two patients are summarized in Table 1. Their family history was unremarkable. Both patients had SNHL since birth and amelogenesis imperfecta on their secondary teeth. Nails, development, and intelligence were normal (Additional file 1: Figure S1 and Additional file 2: Figure S2). They suffered from a progressive decrease in visual acuity and nyctalopia since birth. BCVA values of patient 1 and 2 were 0.8/0.6 and 0.4/0.5, respectively, and intraocular pressures were within the normal range. There were no abnormalities in anterior segments. Fundus examinations revealed midperipheral and peripheral retinal pigmentation abnormalities in both patients, consistent with RP (Figs. 2 and 3). SD-OCT showed marked loss and disruption of the ellipsoid zone, photoreceptor outer segment, and retinal pigment epithelium (RPE). Cystoid macular edema (CME) was seen in both patients, although cystoid spaces were larger and more frequent in patient 2. Interestingly, both patients were previously diagnosed with Usher syndrome as well as attention deficit hyperactivity disorder (ADHD), their axial lengths (20.69 mm/20.49 mm vs 20.17 mm/20.30 mm, respectively) were shorter than their peers, and they suffered from hyperopia (+ 3.25/+ 3.75 DS vs + 3.75/+ 1.65 DS, respectively).

**Table 1:** Summary of mutations, ophthalmologic findings, and other features of the two patients

	Case 1	Case 2
Ethnicity/gender	Chinese/male	Chinese/female
Age of eye examination (years)	9	8
Mutation	*PEX*1 c.2966 T > C; p.Ile989Thr *PEX*1 c.2097_2098insT; p.Phe699Phefs*43	*PEX*1 c.895_896insTATA p.Asn299Ilefs*2 *PEX*1 c.2966 T > C p.Ile989Thr
Family history	Unremarkable	Unremarkable
Ophthalmic findings		
-IOP (mmhg)	10/10	9/10
-BCVA	0.8/0.6	0.4/0.5
- Cycloplegic refraction	R: + 3.25/+ 2.75X85 L: + 3.75/+ 3.00X85	R: + 3.75/− 0.5X67 L: + 1.65/− 0.37X88
- Color amblyopia	Color amblyopia	N
- Cornea	N	N
-Corneal curvature	R: 41.11/45.36 D L: 41.01/44.53 D	R: 45.55/46.30 D L: 45.98/46.49 D
-Central corneal thickness	563 um/563 um	NA
- Anterior chamber depth	2. 65 mm/2.54 mm	1.99 mm/1.99 mm
- Iris	N	N
- Axial lengths	20.69 mm/20.49 mm	20.17 mm/20.30 mm
- Lens	N	N
-UBM	N	N
- Retinal vessels	N	N
-Retina	Retinal atrophy in the mid and far periphery combined with significant bone spicule-like pigmentation	Retinal atrophy in the mid and far periphery combined with significant bone spicule-like pigmentation
- Optic nerve	The inferionasal rim of the optic disc is blurred (R and L)	The inferionasal rim of the optic disc is blurred (R and L)
-B scan	Light to moderate turbidity in the front and middle sections of the vitreous, posterior vitreous detachment	Light turbidity in the front and middle sections of the vitreous, posterior vitreous detachment
-RNFL thickness	N	N
-ffERG	Profoundly attenuated rod ERGs, subnormal and delayed cone ERGs.	Undetectable rod ERG, subnormal bright flash ERG, subnormal and delayed cone ERG.
-mfERG	Amplitudes in posterior-pole of both eyes were significantly reduced, more seriously in the right eye	Amplitudes in posterior-pole of both eyes were significantly reduced, more seriously in the right eye
-VEP	Reduced of P100 amplitude.	Prolongation of latency and lowness of amplitude.
- Humphrey visual field	Peripheral visual field loss, with small central loss	Peripheral visual field loss, except for the superior nasal quadrant
- Goldman visual field	NA	Peripheral visual field constriction
-SD-OCT	Centrally preserved ISe band with small retinal cysts of the inner nuclear layer	Multiple large intraretinal cysts
- Orbital MRI	N	N
- Sensorineural hearing loss	+	+
-Teeth	Amelogenesis imperfecta	Amelogenesis imperfecta
- Nail abnormalities	N	N
- Intellect	N	N
- Electroencephalography	The frequency of theta rhythm is slightly increased	NA
- Development	N	N
- Psychomotor	N	N

Abbreviations. *IOP* intraocular pressure, *BCVA* best corrected visual acuity, *R* right, *L* left, *SD-OCT* spectral domain optical coherence tomography, *UBM* ultrasound biomicroscopy, *RNFL* retinal nerve fiber layer, *MRI* magnetic resonance, *ffERG* full-field ERG, *mfERG* multifocal ERG, *VEP* visual evoked potential, *ISe* inner segment ellipsoid, *N* normal, *NA* not assessed, + positive finding

**Fig. 4:**
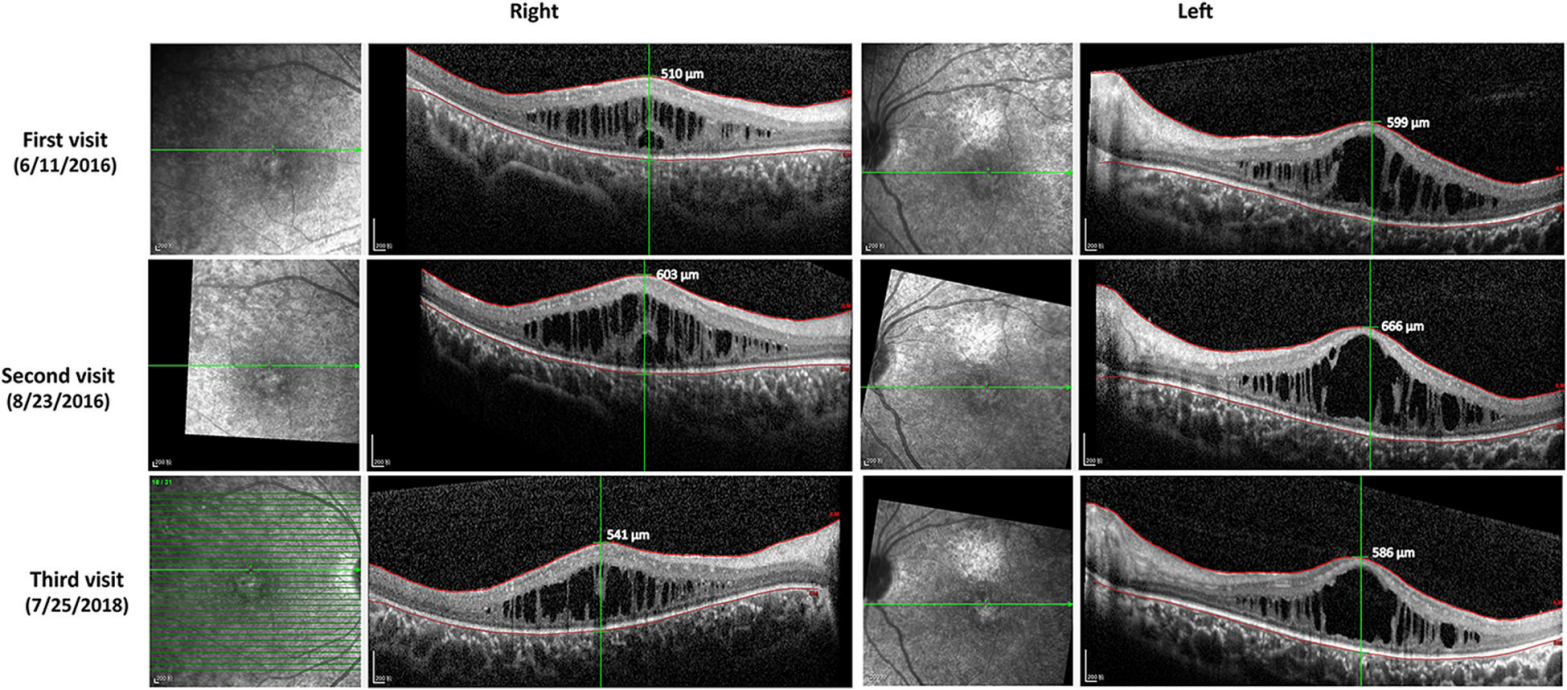
Follow-up of central foveal thickness in patient 2

Follow-up visits were scheduled 2 months and 2 years after the first visit of patient 2. At the 2-month visit, CME was aggravated and the central foveal thickness (CFT) increased from 510 to 603 μm in the right eye and from 599 to 666 μm in the left eye; as a result, the BCVA decreased from 0.6/0.7 to 0.4/0.5. At the third visit 2 years later, the CFT of both eyes had decreased spontaneously; however, through anatomic improvement, there was no notable visual gain (Fig. 4). The thickness of all other retinal layers showed no major change throughout the 2 year-follow-up (Additional file 4: Table S2).

### Genotype–phenotype correlations

Clinical and genetic characteristics of the previously reported HS cases were analyzed and are listed in Table 2. To date, 29 HS patients have been reported worldwide, which gives a total of 31 including the two patients in this study (male = 10, female = 21). Among the 29 reported HS patients, most derive from the UK (*n* = 10) and the USA (*n* = 8), while only one is of Chinese origin. Twelve patients from seven families harbor *PEX6* mutations, 10 patients from eight families carry *PEX1* mutations, and two patients from two families have *PEX26* mutations. No variants were identified in the currently known *PEX* genes in four patients from two families by exome sequencing [9], while genetic testing was undertaken in three patients from three families. Together with our study, 15 missense mutations (71.4%, *PEX1* = 4/8, *PEX6* = 8/10, *PEX26* = 3/3), four frameshift mutations (19.0%, *PEX*1 = 2/8, *PEX6* = 2/10), one nonsense mutation (4.8%, *PEX1* = 1/8), and one splicing mutation (4.8%, *PEX1* = 1/8) have been reported to be associated with HS. Only 50 missense mutations (29.4%, *PEX1* = 21/85, *PEX6* = 27/76, *PEX26* = 2/9) were reported to be associated with Zellweger syndrome, which has a severe early-lethal clinical presentation (Additional file 5: Table S3), while the remaining 70.6% of variants are truncating stop or frameshift defects.

**Table 2:** Phenotype and genotype of individuals with HS reported

Gene	Patients ID	Origin	Sex	Age	Variants	Ocular features	SNHL	Nail	Tooth	Others	Reference
*PEX*1	1F1–1	USA	M	31	c.2114 T > G c.2097dup	RP, MD.	+	Beau’s lines, leukonychia	Amelogenesis imperfecta	N	[1]
	2F1–2	USA	F	29	c.2114 T > G c.2097dup	RP, MD.	+	Beau’s lines, leukonychia	Amelogenesis imperfecta	N	[1]
	9F6–1	Morocco	F	16	c.3750G > A c.3750G > A	N	+	N	Amelogenesis imperfecta	N	[9]
	10F6–2	Morocco	M	12	c.3750G > A c.3750G > A	N	+	N	Amelogenesis imperfecta	N	[9]
	11F7–1	Ireland	F	19	c.1742G > C c.1239 + 1G > T	NA	+	N	Amelogenesis imperfecta	N	[9]
	12F8–1	UK	F	24	c.1742G > C c.2097dup	RP	+	N	Amelogenesis imperfecta	N	[9]
	18F11–1	Moroccan	F	13	c.3140 T > C c.3140 T > C	RP, CME.	+	N	Amelogenesis imperfecta	N	[3]
	27F17–1	China	M	45	c.1792delA c.2966 T > C	Retinal dystrophy	+	Beau’s lines	Amelogenesis imperfecta	Mild learning disability, paranoid schizophrenia, dry split skin on hands; ichthyosis over limbs	[2]
	30F20–1	China	M	9	c.2966 T > C c.2097_2098insT	RP, CME.	+	N	Amelogenesis imperfecta	Hyperactivity, abnormal EEG,θincreases slightly in wake background	This study
	31F21–1	China	F	8	c.2966 T > C c.895_896insTATA	RP, CME.	+	N	Amelogenesis imperfecta	Hyperactivity	This study
*PEX*6	6F4–1	UK	F	21	c.1802G > A c.1841delT	RP	+	Beau’s lines, leukonychia	Amelogenesis imperfecta	N	[12]
	7F4–2	UK	F	21	c.1802G > A c.1841delT	RP	+	Beau’s lines, leukonychia	Amelogenesis imperfecta	N	[12]
	19F12–1	USA	M	NA	c.654C > G c.1802G > A	Retinal dystrophy	NA	NA	Amelogenesis imperfecta	NA	[2]
	20F12–2	USA	F	NA	c.654C > G c.1802G > A	Retinal dystrophy	NA	NA	Amelogenesis imperfecta	NA	[2]
	21F12–3	USA	M	NA	c.654C > G c.1802G > A	Retinal dystrophy	NA	NA	Amelogenesis imperfecta	NA	[2]
	22F13–1	USA	M	12	c.275 T > G c.1802G > A	Retinal dystrophy	+	Beau’s lines	Amelogenesis imperfecta	Hyperpigmentation on left arm and shoulder	[2]
	23F14–1	USA	F	7	c.296G > T c.1802G > A	Retinal dystrophy	+	N	Amelogenesis imperfecta	N	[2]
	24F15–1	UK	F	11	c.1314_1321delGGAGGCCT c.2714G > T	Retinal dystrophy	+	NA	Amelogenesis imperfecta	NA	[2]
	25F16–1	Israel	F	35	c.1715C > T c.1715C > T	Retinal dystrophy	+	N	Amelogenesis imperfecta	Mild learning disability, Light facial lesions	[2]
	26F16–2	Israel	F	22	c.1715C > T c.1715C > T	Retinal dystrophy	+	N	Amelogenesis imperfecta	Mild learning disability, light facial lesions	[2]
	13F9–1	UK	F	21	c.1930C > T c.821C > T	RP	+	Beau’s lines, onychoschizia	Amelogenesis imperfecta	N	[9]
	14F9–2	UK	F	16	c.1930C > T c.821C > T	RP	+	Beau’s lines, onychoschizia	Amelogenesis imperfecta	N	[9]
*PEX*26	28F18–1	Germany	F	4	c.3G > A c.292C > T	RP	+	NA	Amelogenesis imperfecta	NA	[4]
	29F19–1	Germany	M	14	c.127G > C c.292C > T	RP	+	NA	Amelogenesis imperfecta	NA	[4]
–	15F10–1	UK	F	21	–	N	+	N	Amelogenesis imperfecta	N	[9]
	16F10–2	UK	M	20	–	N	+	N	Amelogenesis imperfecta	N	[9]
	17F10–3	UK	M	16	–	N	+	N	Amelogenesis imperfecta	N	[9]
	3F2–1	UK	F	12	–	NA	+	Beau’s lines,	Amelogenesis imperfecta	N	[14]
NA	4F3–1	German and Irish ancestry	F	15	NA	NA	+	Beau’s lines,	Amelogenesis imperfecta	N	[13]
	5F3–2	German and Irish ancestry	F	8	NA	NA	+	Beau’s lines,	Amelogenesis imperfecta	N	[13]
	8F5–1	USA	F	29	NA	RP, CME.	+	Beau’s lines, leukonychia	Amelogenesis imperfecta	N	[11]

Abbreviations. *F* female, *M* male, *N* normal, *NA* not assessed, + positive finding, *SNHL* sensorineural hearing loss, *RP* retinitis pigmentosa, − no variants were identified in currently known *PEX* genes, *MD* macular dystrophy, *CME* cystoid macular edema

**Fig. 5:**
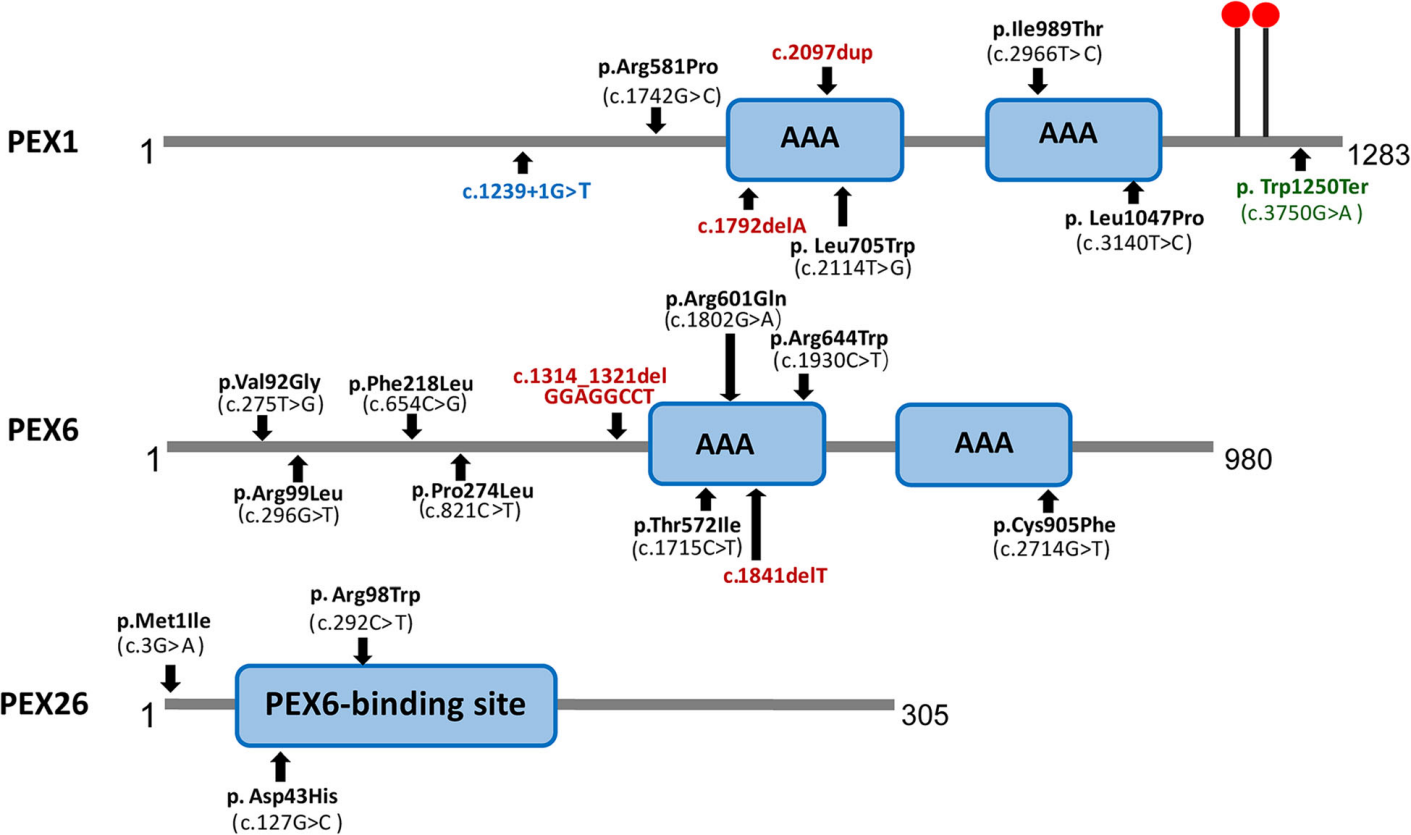
Location of HS-associated variants in *PEX1*, *PEX6* and *PEX26.* Black: missense mutation. Blue: splicing mutation. Red: frameshift mutation. Green: nonsense mutation

The genotype of every HS patient include at least one missense variant, except for two patients from one family with a homozygous exon 23 nonsense variant, c.3750G > A (p.Trp1250*). This variant is only 19 bases from the last exon–exon boundary of *PEX1*, and it is likely that the resultant transcript escapes nonsense-mediated decay with little effect on protein function, resulting in a mild phenotype. We found a genotype–phenotype relationship in HS patients with retinal dystrophy that at least one mutation in *PEX1* or *PEX6* gene affected the nucleotide sequence of the AAA-ATPase region in every patient, which is typically involved in binding of substrates or cofactors and is vital for the *PEX* function^19–21^. These results suggesting that AAA-ATPase region may is a hypermutable region in patients with retinal dystrophy. The locations of these variants are shown in Fig. 5.

All 31 patients were diagnosed with enamel defects of the secondary teeth and SNHL. Twenty patients had RP with or without macular cystoid dystrophy, while no fundus examination was performed on the other four patients so it is unknown if they had RP. Funduscopy of five patients from two families was normal. Twelve patients have nail abnormalities, suggesting that this is not an essential phenotype so should not be a clinical diagnostic indicator for HS [12]. HS was shown to be a rare disease with high phenotypic heterogeneity, with no significant difference between *PEX1-*, *PEX6-*, and *PEX26*-associated phenotypes.

## Discussion

Phenotypic information about individual patients is often insufficiently detailed or inaccessible, thus obstructing the diagnosis of rare systemic diseases or those with overlapping phenotypes. Gene panel sequencing achieves high diagnostic rates in the context of a specific suspected disease or group of diseases, and genetic results can provide support for clinical diagnosis, modify future disease risk, and inform the customization of a variety of therapies [16, 17].

So far, our clinical knowledge and understanding of HS are very limited and a diagnosis can easily be overlooked or misdiagnosed. In this work, two patients previously diagnosed with Usher syndrome achieved an accurate diagnosis of HS based on NGS. Additionally, a novel pathogenic mutation and two unreported ocular phenotypes were recognized, and four novel genotype–phenotype correlations of HS were uncovered. This provides a deeper understanding of the clinical manifestations and pathogenesis of HS, which is vital for accurate clinical practice, genetic counseling, and pathogenesis studies.

No details of HS ophthalmic manifestations caused by *PEX1* mutations have been reported so far. In this work, we present comprehensive ophthalmic examinations of two HS patients induced by *PEX1* mutations. As well as the previously reported phenotypic features of RP with CME, these patients also showed short axial lengths and hyperopia suggesting that *PEX1* mutation-associated ocular involvement can be developmental (short axial length) and degenerative (RP). Both patients also had ADHD which may be a novel phenotype of HS. After comprehensively analyzing the varied phenotypes of all reported HS cases in the literature, we propose that HS is a systemic disorder with high phenotypic variability; however, all cases demonstrate SNHL (severe to profound) and enamel hypoplasia of secondary dentition. Most patients show infantile-onset retinal dystrophy, and nails are normal or abnormal. They may also suffer from other systemic abnormalities such as learning disabilities or ADHD. Considering the wide clinical heterogeneity, rapid and definitive diagnosis of HS is challenging, and it is especially difficult to establish clinical diagnostic criteria. NGS can help resolve these challenges, and we suggest that it should become the “gold standard” for diagnosing HS.

Through a comprehensive analysis of the reported mutant genes we found that mutations in *PEX1* and *PEX6* are the main cause of HS. A small number of cases are also caused by mutations in *PEX26*, and it is likely that other HS-causing genes remain to be discovered. *PEX1* and *PEX6* are members of the AAA protein family that are involved in binding ATP to form a heterohexameric ATPase which is associated with various cellular activities that fuel essential protein transport across peroxisomal membranes [20, 21]. They also play a key role in matrix protein import from heterohexamers and ternary complexes with *PEX26* [22]. Mutant *PEX6* and *PEX1* proteins result in abnormal peroxisomal function, leading to the accumulation of very long chain fatty acids in photoreceptors, RPE, and pigment-laden macrophages [10, 23].

Genotype–phenotype analysis in this study revealed that HS is caused by genotypes including at least one missense variant, while Zellweger syndrome was caused by more deleterious genotypes, such as truncating stop or frameshift defects. Furthermore, at least one mutation in one patient affects the AAA-ATPase region (*PEX1* and *PEX6*) or *PEX6* binding site (*PEX26*) when HS patients have retinal dystrophy. Together, these results we speculate that hypomorphic mutations in *PEX* result in a partially functional protein [24, 25], mutations in AAA-ATPases play a role in retinal dystrophy, and AAA-ATPase region is a hypermutable region in patients with a retinal dystrophy. No significant difference was found among *PEX1*-, *PEX6*-, and *PEX26*-associated phenotypes, perhaps because these genes closely interact in vivo so any mutation in a given gene affects the function of the entire complex [26, 27]. It is also conceivable that a single genetic locus cannot explain the full phenotypic spectrum, and we propose that additional genetic and possibly nongenetic modifiers cause the various phenotypes.

One limitation of this study is that the genotype–phenotype correlations were based on only 31 HS patients. Therefore, these correlations should be confirmed in more HS patients in future studies. Additionally, only one HS patient was followed up over a 2-year period, which may not accurately reflect the progress of the disease. Longer follow-up in more HS patients is required to better understand the ocular prognosis of this disease.

## Conclusions

To summarize, we report two HS families diagnosed by NGS and present the first comprehensive report of the HS ophthalmic phenotype. We identified a novel pathogenic mutation and two unreported ocular phenotypes, which expands the known mutation and clinical spectra of HS. Furthermore, we propose that HS is a systemic disorder with high phenotypic heterogeneity, and show the importance of NGS in the diagnosis of HS. Three novel genotype–phenotype correlations of HS were also uncovered, which is useful for accurate clinical practice, genetic counseling, and pathogenesis studies. These data should be validated and expanded in additional studies.

## Supplementary information

**Additional file 1: Figure S1.** Clinical characteristics of patient 1. A and B: the hearing test demonstrated the presence of sensorineural hearing loss. C and D: The clinical appearance of teeth and orthopantograms show severe amelogenesis imperfecta. E: Fingernails and toenails are normal, and no Beau’s lines or leukonychia were observed.

**Additional file 2: Figure S2.** Clinical characteristics of patient 2. A and B: The hearing test shows that both ears suffered from SNHL since birth. C and D: The clinical appearance of teeth and orthopantograms show severe amelogenesis imperfecta. E: Fingernails and toenails are normal, and no Beau’s lines or leukonychia were observed.

**Additional file 3: Table S1.** Gene list of capture panel (762 genes).

**Additional file 4: Table S2**. Follow-up of intraretinal layer thickness in patient 2.

**Additional file 5: Table S3.** Reported pathogenic variants in *PEX1*, *PEX6*, and *PEX26* in the Human Gene Mutation Database associated with Zellweger syndrome.

## Data Availability

Please contact authors for data requests.

## Abbreviations

ACMG: American College of Medical Genetics
ADHD: Attention deficit hyperactivity disorder
ATP: Adenosine triphosphate
BCVA: Best corrected visual acuity testing
CFT: Central foveal thickness
CME: Cystoid macular edema
ERG: Full-field electroretinography
HGMD: Human Gene Mutation Database
HS: Heimler syndrome
IOP: Intraocular pressure
NGS: Next-generation sequencing
OMIM: Online Mendelian Inheritance in Man
PBDs: Peroxisome biogenesis disorders
RP: Retinitis pigmentosa
RPE: Retinal pigment epithelium
SD-OCT: Spectral domain optical coherence tomography
SNHL: Sensorineural hearing loss
UBM: Ultrasound biomicroscopy
UTRs: Untranslated regions
