# Supplementary figures and images for: Expanding the clinical and genetic spectrum of Heimler syndrome
Source: Orphanet J Rare Dis. 2019 Dec 12;14:290. doi: 10.1186/s13023-019-1243-x (PMC6909578; doi:10.1186/s13023-019-1243-x)

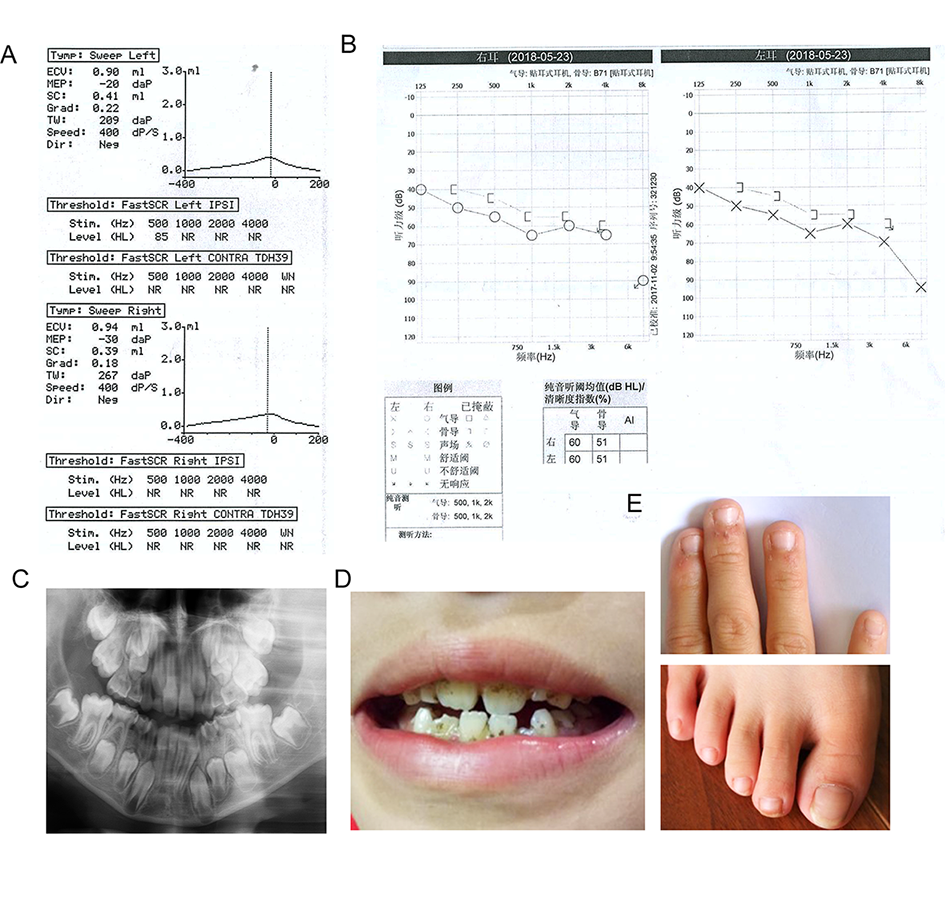

Supplement: Supplementary file 1 — Additional file 1: Figure S1. Clinical characteristics of patient 1. A and B: the hearing test demonstrated the presence of sensorineural hearing loss. C and D: The clinical appearance of teeth and orthopantograms show severe amelogenesis imperfecta. E: Fingernails and toenails are normal, and no Beau’s lines or leukonychia were observed. [file 13023_2019_1243_MOESM1_ESM.tif]

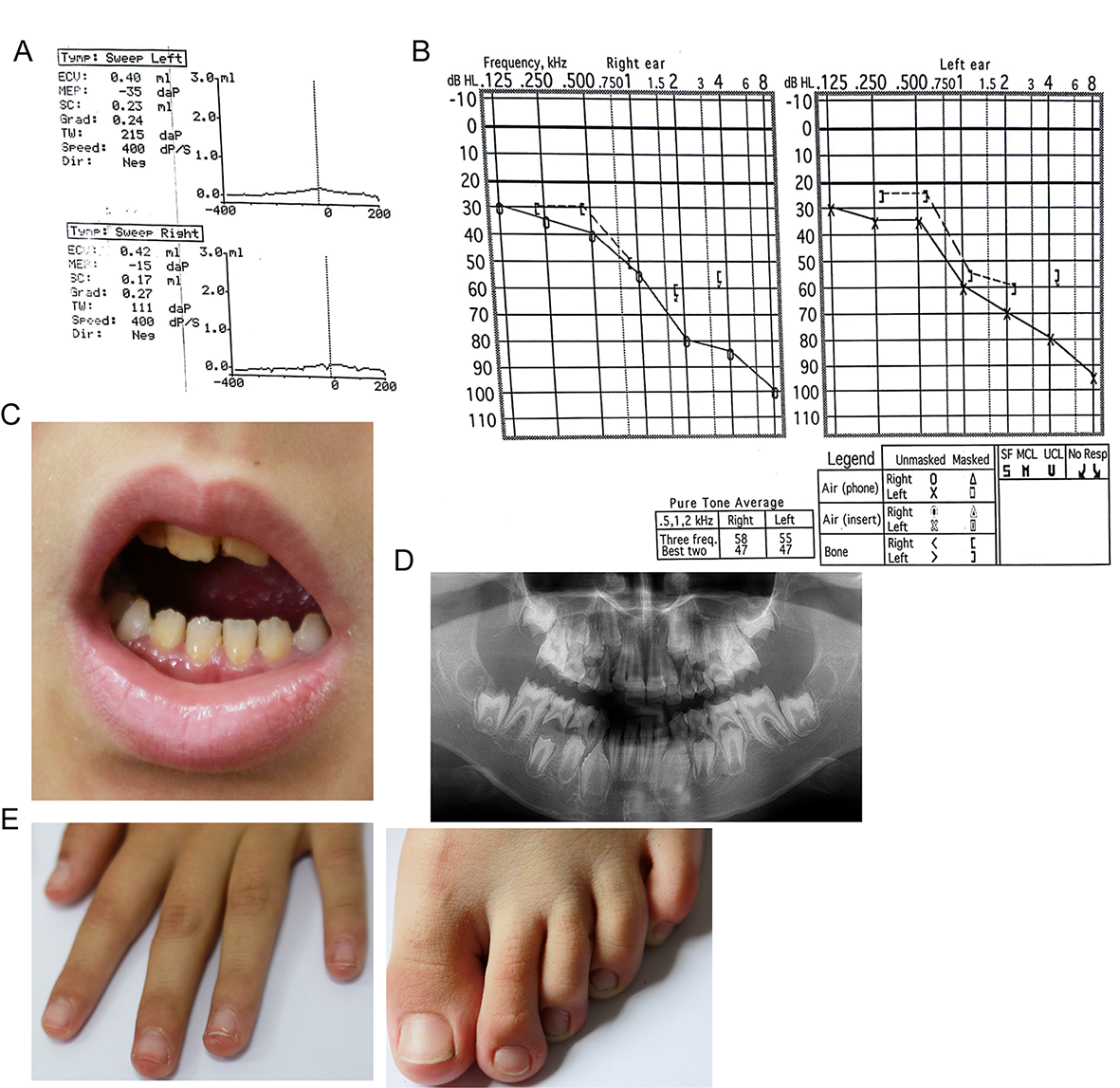

Supplement: Supplementary file 2 — Additional file 2: Figure S2. Clinical characteristics of patient 2. A and B: The hearing test shows that both ears suffered from SNHL since birth. C and D: The clinical appearance of teeth and orthopantograms show severe amelogenesis imperfecta. E: Fingernails and toenails are normal, and no Beau’s lines or leukonychia were observed. [file 13023_2019_1243_MOESM2_ESM.tif]
