# Supplementary material for: Expanding the clinical and genetic spectrum of Heimler syndrome
Source: Orphanet J Rare Dis. 2019 Dec 12;14:290. doi: 10.1186/s13023-019-1243-x (PMC6909578; doi:10.1186/s13023-019-1243-x)
Supplement: Supplementary file 3 — Additional file 3: Table S1. Gene list of capture panel (762 genes). [file 13023_2019_1243_MOESM3_ESM.pdf]

Gene	OMIM	Gene	OMIM	Gene	OMIM	Gene	OMIM
GJA8	600897	LYST	606897	NDP	300658	BCOR	300485
CRYBA1	123610	ERCC8	609412	RP1L1	608581	SOX2	184429
PITX3	602669	LONP1	605490	CTNNA1	116805	OTX2	600037
BFSP2	603212	PIGL	605947	MT-TH	590040	BMP4	112262
GCNT2	600429	CTDP1	604927	TIMP3	188826	HCCS	300056
GJA3	121015	FBN2	612570	ABCC6	177850	TTR	105210
MIP	154050	POMT1	607423	RDH5	601617	TCOF1	606847
CRYAB	123590	TMEM5	605862	WDR19	608151	TUBB3	602661
CRYBB1	600929	B3GALNT2	610194	KCNV2	607604	VHL	608537
FYCO1	607182	POMK	615247	CACNA2D4	608171	SMOC1	608488
LIM2	154045	GMPPB	615320	CLCN7	602727	TYR	606933
CRYGC	123680	POMT2	607439	HFE	613609	PAX3	606597
CRYGS	123730	POMGNT1	606822	ASRGL1	609212	MITF	156845
MAF	177075	FKTN	607440	CEP164	614848	SNAI2	602150
CRYBB3	123630	FKRP	606596	GFAP	137780	EDNRB	131244
CRYBA4	123631	ISPD	614631	MIR204	610942	EDN3	131242
CRYBB2	123620	POMGNT2	614828	RDH11	607849	SOX10	602229
VIM	193060	PLG	173350	TUB	601197	RAB3GAP1	602536
CHMP4B	610897	TIMM8A	300356	ITM2B	603904	RAB3GAP2	609275
BFSP1	603307	EP300	602700	RBP4	180250	RAB18	602207
TDRD7	611258	FUCA1	612280	LAMA1	150320	TBC1D20	611663
AGK	610345	ITGB3	173470	PANK2	606157	WRN	604611
CRYGB	123670	CHN1	118423	PRKCG	176980	ATP7B	606882
CRYGD	123690	SALL4	607343	TRNT1	612907	NIPBL	608667
NHS	300457	ABCC2	601107	ABHD12	613599	HDAC8	300269
WFS1	606201	ACD	609377	MT-TP	590075	GPR143	300808
CRYBA2	600836	NHP2	606470	ANAPC1	608473	DNAJC19	608977
HSF4	602438	NOP10	606471	EMC1	616846	SF3B4	605593
EPHA2	176946	TERT	187270	NEUROD1	601724	PIK3R1	171833
CRYAA	123580	WRAP53	612661	OR2W3	616729	HGD	607474
IARS2	612801	EDARADD	606603	SPP2	602637	KCNJ10	602208
MSMO1	607545	PKP1	601975	ADGRA3	612303	MRE11A	600814
CAV1	601047	COL3A1	120180	AGBL5	615900	ATM	607585
SLC33A1	603690	PLOD1	153454	ARL3	604695	ATP2C1	604384
FOXE3	601094	TNXB	600985	DHX38	605584	COL6A3	120250
ADAMTSL4	610113	LOXL1	153456	EXOSC2	602238	COL6A2	120240
SLC16A12	611910	MC1R	155555	HK1	142600	BLM	210900
FBN1	134797	IKBKAP	603722	KIAA1549	613344	MTM1	300415
ADAMTS10	608990	FGFR1	136350	KIZ	615757	CYP27A1	606530
ADAMTS17	607511	FGFR2	176943	MVK	251170	TFAP2B	601601
DCN	125255	FGFR3	134934	PRPF4	607795	DNM2	602378
KERA	603288	LCAT	606967	FLVCR1	609144	PDK3	300906
TGFBI	601692	FRAS1	607830	RP1	603937	ARSE	300180
TACSTD2	137290	GRIP1	601993	IMPDH1	146690	EBP	300205
KRT12	601687	FREM2	608945	PRPF31	606419	GNPAT	602744
VSX1	605020	FRMD7	300628	CRB1	604210	CNTN1	600016
SLC4A11	610206	GALK1	604313	PRPF8	607300	RAD21	606462
PIKFYVE	609414	GALE	606953	TULP1	602280	SMC1A	300040
PXDN	605158	GALT	606999	CA4	114760	ALDH18A1	138250
COL8A2	120252	ANTXR1	606410	PRPF3	607301	ATP6V0A2	611716

TCF4	602272	GBA	606463	ABCA4	601691	EFEMP2	604633
ZEB1	189909	ROBO3	608630	RP2	300757	LTBP4	604710
CHST6	605294	GLB1	611458	RPE65	180069	PYCR1	179035
KRT3	148043	GM2A	613109	OFD1	300170	PRX	605725
CHRDL1	300350	HEXA	606869	EYS	612424	DBH	609312
UBIAD1	611632	MLPH	606526	CERKL	608381	ADAR	146920
ASB10	615054	RAB27A	603868	NRL	162080	EMD	300384
MYOC	601652	JAM3	606871	FAM161A	613596	LMNA	150330
OPTN	602432	ACVRL1	601284	RPGR	312610	SYNE2	608442
WDR36	609669	ATL1	606439	FSCN2	613596	LAMA3	600805
NTF4	162662	HPS1	604982	TOPORS	609507	MYH11	160745
CYP1B1	601771	AP3B1	603401	SNRNP200	601664	MYLK	600922
LTBP2	602091	HPS3	606118	SEMA4A	607292	PRKG1	176894
ATF6	605537	HPS4	606682	PRCD	610598	ASAH1	613468
CNGA3	600053	HPS5	607521	NR2E3	604485	PORCN	300651
CNGB3	605080	HPS6	607522	MERTK	604705	FMR1	309550
GNAT2	139340	DTNBP1	607145	RHO	180380	CTSA	613111
PDE6H	601190	BLOC1S3	609762	PDE6B	180072	APC	611731
HMCN1	608548	BLOC1S6	604310	PROM1	604365	KIF1BP	609367
TLR4	603030	CBS	613381	KLHL7	611119	AGXT	604285
CST3	604312	IDUA	252800	PDE6A	180071	GRHPR	604296
CX3CR1	601470	FTL	134790	RGR	600342	HOGA1	613597
CFI	217030	GCM2	603716	CNGB1	600724	PTH	168450
C2	613927	FAM126A	610531	IDH3B	604526	STS	300747
CFB	138470	GJB2	121011	SAG	181031	KDM6A	300128
C9	120940	IKBKG	300248	GUCA1B	602275	KMT2D	602113
FBLN5	604580	RAX	601881	CNGA1	123825	ATP13A2	610513
CFH	134370	GDF6	601147	TTC8	608132	SGCB	600900
ERCC6	609413	MFRP	606227	C2orf71	613425	SGCD	601411
HTRA1	602194	PRSS56	613858	ARL6	608845	TTN	188840
ARMS2	611313	GDF3	606522	IMPG2	607056	COX7B	300885
C3	120700	KIF21A	608283	PDE6G	180073	CREBBP	600140
ALMS1	606844	COL18A1	120328	ZNF513	613598	TGFBR1	190181
GABRB1	137190	GALC	606890	DHDDS	608172	TGFBR2	190182
IFT27	615870	COX10	602125	PRPF6	613979	SMAD3	603109
BBS1	209901	COX15	603646	CLRN1	606397	TGFB2	190220
BBS10	610148	SURF1	185620	MAK	154235	TGFB3	190230
TRIM32	602290	OCRL	300535	C8orf37	614477	KCNH2	152427
BBS12	610683	FREM1	608944	CDHR1	609502	KCNJ2	600681
MKS1	609883	MAP2K1	176872	RBP3	180290	TSC1	605284
WDPCP	613580	SIL1	608005	NEK2	604043	TSC2	191092
SDCCAG8	613524	TUBGCP4	609610	SLC7A14	615720	MAN2B1	609458
LZTFL1	606568	TUBGCP6	610053	PRPH2	179605	MANBA	609489
BBIP1	613605	VSX2	142993	IFT172	607386	GNAS	139320
BBS2	606151	SHH	600725	HGSNAT	610453	FLNA	300017
BBS4	600374	ABCB6	605452	RP9	607331	AUH	600529
BBS5	603650	STRA6	610745	GNPTG	607838	MMACHC	609831
MKKS	604896	ALDH1A3	600463	ARL2BP	615407	ASPM	605481
BBS7	607590	DHODH	126064	INVS	243305	CDK5RAP2	608201
BBS9	607968	RYR1	180901	NPHP1	607100	CEP135	611423
PLA2G5	601192	GFER	600924	NPHP4	607215	CEP152	613529

OPN1MW	300822	ACTA2	102620	IQCB1	609237	ZNF335	610827
RLBP1	180090	TRIM37	605073	ELOVL4	605512	TACO1	612958
PNPLA6	603197	B4GAT1	605517	TEAD1	189967	DYSF	603009
KIF11	148760	DAG1	128239	OPN1SW	613522	MCOLN1	605248
CHM	303100	LARGE1	603590	ADGRV1	602851	ARSB	611542
TMEM67	609884	AGRN	103320	WHRN	607928	GALNS	612222
OPN1LW	300824	CHAT	118490	CEP250	609689	GNS	607664
DRAM2	613360	COLQ	603033	MYO7A	276903	GUSB	611499
PCYT1A	123695	ADAMTS18	607512	USH1C	605242	IDS	300823
UNC119	604011	LAMB2	150325	CDH23	605516	NAGLU	609701
C21orf2	603191	PPT1	600722	PCDH15	605514	MSH2	609309
RAX2	610362	CTSD	116840	USH1G	607696	ITGA7	600536
GUCA1A	600364	GRN	138945	USH2A	608400	LAMA2	156225
RAB28	612994	CTSF	603539	PDZD7	612971	SEPN1	606210
TTLL5	612268	TPP1	607998	HARS	142810	PLEC	601282
POC1B	614784	CLN3	607042	CIB2	605564	CHRNG	100730
PDE6C	600827	DNAJC5	611203	MT-TS2	590085	DOK7	610285
PITPNM3	608921	CLN6	608102	VCAN	118661	MSTN	601788
RIMS1	606629	CLN5	608102	TLR2	603028	SCN4A	603967
ADAM9	602713	CLN8	607837	RS1	300839	DMPK	605377
ACBD5	616618	MT-ATP6	516060	PEX10	602859	CNBP	116955
GNAT1	139330	ABHD5	275630	PEX11B	603867	LMX1B	602575
GRK1	180381	NPC2	601015	PEX12	601758	NPC1	607107
CACNA1F	300110	SMPD1	607608	PEX13	601789	TPM2	190990
NYX	300278	PTPN11	176876	PEX14	601791	TPM3	191030
TRPM1	603576	KRAS	190070	PEX16	603360	NEB	256030
SLC24A1	603617	SOS1	182530	PEX26	608666	CFL2	601443
GRM6	604096	RAF1	164760	PEX5	600414	SPINK5	605010
CABP4	608965	NRAS	164790	PEX6	601498	PLA2G6	603604
GPR179	614515	BRAF	164757	PEX2	170993	C19orf12	614297
LRIT3	615004	IGBP1	300139	PEX7	601757	COASY	609855
GNB3	139130	HMX1	142992	SLITRK6	609681	MID1	300552
CYP4V2	608614	OCA2	611409	P3H2	610341	DDX59	615464
ROM1	180721	TYRP1	115501	LRPAP1	104225	TNFRSF11A	603499
EFEMP1	601548	SLC45A2	606574	PRIMPOL	615421	OSTM1	607649
ZNF408	616454	SLC24A5	609802	ZNF644	614159	SNX10	614780
RCBTB1	607867	C10orf11	614537	ACO2	100850	TCIRG1	604592
FZD4	604579	GJA1	121014	MT-ND1	516000	TNFSF11	602642
LRP5	603506	PABPN1	602279	ATP1A3	182350	SLC26A4	605646
TSPAN12	613138	KCTD7	611725	RTN4IP1	610502	STK11	602216
OAT	613349	DMD	300377	UCHL1	191342	PGK1	311800
CNNM4	607805	B3GLCT	610308	AFG3L2	604581	KIT	164920
CSPP1	611654	PHOX2A	602753	C12orf65	613541	CHMP1A	164010
TCTN2	613846	TUBA8	605742	PRPS1	311850	EXOSC3	606489
B9D1	614144	POLG	174763	SLC25A46	610826	RARS2	611524
INPP5E	613037	RNASEH1	604123	OPA1	605290	TSEN2	608753
TTC21B	612014	SLC25A4	103220	OPA3	606580	TSEN34	608754
KIF7	611254	C10orf2	606075	TMEM126A	612988	TSEN54	608755
TCTN1	609863	POLG2	604983	MFN2	608507	VRK1	602168
TMEM237	614423	RRM2B	604712	SIX6	606326	UROD	613521
CEP41	610523	DNA2	601810	NBAS	608025	PHYH	602026

TMEM138	614459	RGS9	604067	PAX6	607108	AGPS	603051
C5orf42	614571	RGS9BP	607814	GP1BA	606672	TWIST1	601622
TCTN3	613847	SLC4A4	603345	CISD2	611507	ACTA1	102610
ZNF423	604557	PAX2	167409	NF2	607379	FHL1	300163
TMEM216	613277	ZFYVE26	612012	NF1	162200	TRPV4	605427
TMEM231	614949	TREX1	606609	COLEC11	612502	HSPG2	142461
AHI1	608894	PLK4	605031	MPZ	159440	NEU1	608272
ARL13B	608922	TINF2	604319	JAG1	601920	DHCR7	602858
CC2D2A	612013	KIAA0196	610657	NOTCH2	600275	NFIX	164005
LRAT	604863	RECQL4	268400	COL4A4	120131	AP4M1	602296
C1QTNF5	608752	ESCO2	609353	COL4A5	303630	AP5Z1	613653
CLUAP1	616787	HESX1	601802	TP63	603273	B4GALNT1	601873
NPHP3	608002	ALDH3A2	609523	FGF10	602115	CYP2U1	610670
PEX1	602136	MTPAP	613669	SLC2A10	606145	CYP7B1	603711
GUCY2D	600179	SYNE1	608441	APTX	606350	DDHD2	615003
CEP290	610142	ANO10	613726	SETX	608465	ERLIN2	611605
RD3	180040	EEF2	130610	FOXC1	601090	FA2H	611026
RDH12	608830	ITPR1	147265	PITX2	601542	GBA2	609471
KCNJ13	603208	KCNC3	176264	ACTB	102630	HSPD1	118190
SPATA7	609868	KCND3	605411	ACTG1	102560	KIF1A	601255
AIPL1	604392	SPTBN2	604985	PTCH1	601309	KIF5A	602821
LCA5	611408	SYT14	610949	PTCH2	603673	MARS2	609728
RPGRIP1	605446	TGM6	613900	NSD1	606681	NIPA1	608145
CRX	602225	TTBK2	611695	NOD2	605956	REEP1	609139
NMNAT1	608700	WWOX	605131	FOXL2	605597	RTN2	603183
DTHD1	616979	ATXN10	611150	DRD5	126453	SPG11	610844
ADIPOR1	607945	ATXN7	607640	PHF6	300414	SPG7	602783
MT-TL1	590050	COL2A1	120140	NR2F1	132890	ZFYVE27	610243
SOD2	147460	COL11A1	120280	COL4A1	120130	MAPT	157140
BEST1	607854	COL11A2	120290	TFAP2A	107580	SALL1	602218
IMPG1	602870	COL9A1	120210	ZNF469	612078	POLR1C	610060
PRDM13	616741	COL9A2	120260	PRDM5	614161	BAP1	603089
CDH3	114021	NAA10	300013	CYLD	605018	FAH	613871
IFT140	614620	VAX1	604294	ABCA3	601615	IRF6	607199
MAPKAPK3	602130	RARB	180220	CCM2	607929	TBX1	602054
CAPN5	602537	HMGB3	300193	ERCC2	126340	EPG5	615068
MFSD8	611124	MAB21L2	604357	ERCC1	126380	ERCC3	133510
SEMA3E	608166	RB1	614041	CTC1	613129	XPA	611153
CHD7	608892	XPC	613208				
