# Supplementary material for: Expanding the clinical and genetic spectrum of Heimler syndrome
Source: Orphanet J Rare Dis. 2019 Dec 12;14:290. doi: 10.1186/s13023-019-1243-x (PMC6909578; doi:10.1186/s13023-019-1243-x)
Supplement: Supplementary file 4 — Additional file 4: Table S2. Follow-up of intraretinal layer thickness in patient 2. [file 13023_2019_1243_MOESM4_ESM.pdf]

Supplementary Table 2 Follow-up of intraretinal layer thickness in patient 2.

Thickness (μm)	First visit	2 months	2 years
TR	510/599	603/666	541/586
RNFL	14/14	14/13	13/13
GCC	9/8	8/8	7/8
IPL	15/12	15/10	15/10
INL	15/10	14/8	14/8
OPL	30/18	30/12	29/13
ONL	299/426	412/513	324/415
RPE	13/11	13/11	10/11

Abbreviations. TR: total retinal, RNFL: retinal nerve fiber layer, GCL: ganglion cell layer, IPL: inner plexiform layer, INL: inner nuclear layer, OPL: outer plexiform layer, ONL: outer nuclear layer, RPE: retinal pigment epithelium.
