# Supplementary material for: Expanding the clinical and genetic spectrum of Heimler syndrome
Source: Orphanet J Rare Dis. 2019 Dec 12;14:290. doi: 10.1186/s13023-019-1243-x (PMC6909578; doi:10.1186/s13023-019-1243-x)
Supplement: Supplementary file 5 — Additional file 5: Table S3. Reported pathogenic variants in PEX1, PEX6, and PEX26 in the Human Gene Mutation Database associated with Zellweger syndrome. [file 13023_2019_1243_MOESM5_ESM.pdf]

Supplementary table 3. Reported pathogenic variants in the PEX1, PEX6 and PEX26 genes in HGMD associated with Zellweger syndrome.

Gene	Nucleotide	Amino acid change
PEX6	c.277C>G	Arg93Gly
PEX6	c.280G>C	Ala94Pro
PEX6	c.557C>A	Ala186Glu
PEX6	c.611C>G	Ser204Term
PEX6	c.656A>C	Gln219Pro
PEX6	c.659G>T	Gly220Val
PEX6	c.1198T>A	Tyr400Asn
PEX6	c.1532T>G	Leu511Arg
PEX6	c.1553C>A	Ala518Asp
PEX6	c.1711G>A	Ala571Thr
PEX6	c.1801C>T	Arg601Trp
PEX6	c.1814T>G	Leu605Arg
PEX6	c.1958C>G	Ser653Term
PEX6	c.1992G>C	Glu664Asp
PEX6	c.2225T>C	Leu742Pro
PEX6	c.2356C>T	Arg786Trp
PEX6	c.2362G>A	Val788Met
PEX6	c.2440C>T	Arg814Term
PEX6	c.2482C>T	Gln828Term
PEX6	c.2591T>C	Phe864Ser
PEX6	c.2626C>T	Arg876Trp
PEX6	c.2663G>C	Arg888Pro
PEX6	c.2726T>A	Leu909Gln
PEX6	c.2735C>T	Ala912Val
PEX6	c.1047-2A>G	
PEX6	c.1047-1G>A	
PEX6	c.1054C>T	
PEX6	c.1131-1G>C	
PEX6	c.1689-1G>T	
PEX6	c.2095-2A>G	
PEX6	c.2301-15C>G	
PEX6	c.2301-5C>G	
PEX6	c.2300+28G>A	
PEX6	c.2667-2A>G	
PEX6	c.2807-2A>G	
PEX6	c.115_120delGCCCTG	
PEX6	c.311delG	
PEX6	c.656delA	
PEX6	c.689_690delAG	
PEX6	c.856delC	
PEX6	c.867delA	
PEX6	c.1314_1321delGGAGGCCT	
PEX6	c.1367+1delG	
PEX6	c.1404delA	
PEX6	c.1495delC	
PEX6	c.1793_1794delAG	
PEX6	c.1947delG	
PEX6	c.2207_2217del11	
PEX6	c.224dupT	
PEX6	c.1313dupT	
PEX6	c.883_1130del248	
PEX6	ex. 14-16	
PEX6	c.2602_2603ins350	

PEX6	c.10_69del;c.126_216del, p.A4_L23del;p.A43fsX31	
PEX6	c.727C>T	Gln243Term
PEX6	c.1646C>T	Ala549Val
PEX6	c.1802G>A	Arg601Gln
PEX6	c.2435G>A	Arg812Gln
PEX6	c.2434C>T	Arg812Trp
PEX6	c.2546A>C	Asn849Thr
PEX6	c.2579G>A	Arg860Gln
PEX6	c.2578C>T	Arg860Trp
PEX6	c.883-2A>G	IVS1 as A-G -2
PEX6	c.1130+1G>A	IVS3 ds G-A +1
PEX6	c.1688+1G>A	IVS7 ds G-A +1
PEX6	c.2666+2T>C	IVS15 ds T-C +2
PEX6	c.275_280delTGCGGG	
PEX6	c.402delC	
PEX6	c.530delC	
PEX6	c.914delA	
PEX6	c.1301delC	
PEX6	c.510dupT	TCCAGAG^170TCTtGGGGACAGCA
PEX6	c.689_690dupAG	GGCCAGA^230GAGagTCATCGAACA
PEX6	c.814_817dupCTTG	CTGGCG^272CTTGcttgTCCCTGCCAC
PEX6	c.1415dupC	TACGG^470GGCCCcCCCAGGCTGT
PEX6	c.1891delG	CTAG_IE_GGC^630TTTgTGGTAGGGGA
PEX1	c.911_912delCT	TCAGCA^303ACCTctGTTTTTCATA
PEX1	c.782_783delAA	GAGAAG^260AAACaaGAGACATCTT
PEX1	c.781C>T	Gln261Term
PEX1	c.760dupT	AGCATT^253TTTTtCCTTTCAATC
PEX1	c.643_647delACCAA	AGAACTT^214CAAaccaaGCAACTTCAG
PEX1	c.627G>A	Met209Ile
PEX1	c.616C>T	Gln206Term
PEX1	c.5G>A	Trp2Term
PEX1	c.569C>A	Ser190Term
PEX1	c.56_57delTG	GCAGTG^18ACTGtgGCCTTCACCA
PEX1	c.547C>T	Arg183Term
PEX1	c.475G>C	Ala159Pro
PEX1	c.473-1G>A	IVS4 as G-A -1
PEX1	c.3G>A	Met1Ile
PEX1	c.3732_3736dupCATTA	CCATCC^1245ATTAcattaGTGAAGATGA
PEX1	c.3710C>A	Ala1237Glu
PEX1	c.3693_3696delGTCA	GGCTATT^1230AGTcagtCACATTTAAT
PEX1	c.3438+2T>C	IVS21 ds T-C +2
PEX1	c.343_344insCT	TCAGCA^114GATGctATTGGGAGAT
PEX1	c.3379dupC	AATATG^1126TACCcGGCTCTACTT
PEX1	c.3378C>G	Tyr1126Term
PEX1	c.3287C>G	Ser1096Term
PEX1	c.3180dupT	GGCCTTA^1060CATtGGAATGCTGC
PEX1	c.3038G>A	Arg1013His
PEX1	c.3030G>T	Gln1010His
PEX1	c.3022_3024delCCT	CTGTCCT^1007CCTcctGATCAG_E19I19_GTGA
PEX1	c.2T>C	Met1Thr
PEX1	c.2927-15_3208-342delinsATAGTATAGA,c.3849+779_3849+2910del	
PEX1	c.2926+2dupT	CTTA^975CAGG_E18I18_GTtAATAATTATA
PEX1	c.2916delA	ATGGA^971GTAGAaGGCTTACAGG
PEX1	c.2846G>A	Arg949Gln

PEX1	c.2845C>T	Arg949Trp
PEX1	c.2843G>A	Arg948Gln
PEX1	c.2814_2818delCTTTG	GCATT^937CTTTTctttgATGAATTTGA
PEX1	c.2760delA	CAAGT^919GAACAaGCTGTTCGGG
PEX1	c.274G>C	Val92Leu
PEX1	c.2730delA	GGCCA^909GAGTTaCTCAGCAAAT
PEX1	c.273+1G>A	IVS2 ds G-A +1
PEX1	c.270delA	CAAAT^89GGGGGaCAG_E2I2_GTAAGCA
PEX1	c.2654C>G	Thr885Arg
PEX1	c.2636T>C	Leu879Ser
PEX1	c.2636_2638delTGT	ACAGGA^877ATACtgtTGTATGGTCC
PEX1	c.2614C>T	Arg872Term
PEX1	c.2537_2545delATGAAGTTAinsTC	GGTGGG^845TTACatgaagttaGGCAGATACT
PEX1	c.2489delA	CGAAGT^829GTCAaCCTGCATAAA
PEX1	c.2479C>T	Arg827Term
PEX1	c.2416+1G>T	IVS14 ds G-T +1
PEX1	c.2391_2392delTC	CTCGA^796CTCTCtcGTCAGAGTAT
PEX1	c.2387T>C	Leu796Pro
PEX1	c.2383C>T	Arg795Term
PEX1	c.2364G>A	IVS14 ds G-A -53
PEX1	c.2224C>T	Gln742Term
PEX1	c.2097dupT	TAAAA^698GAGTTtTATCTCCATG
PEX1	c.2085_2089delGATAA	TGAAT^694GATATgataaAAGAGTTTAT
PEX1	c.2085_2087delGAT	TTTGAAT^694GATatgATAAAAGAGT
PEX1	c.2034_2035delCA	AACAT^677GAGCAcaGTCCTGATGC
PEX1	c.1991T>C	Leu664Pro
PEX1	c.1976T>A	Val659Asp
PEX1	c.1957T>A	Trp653Arg
PEX1	c.1900+2T>C	IVS11 ds T-C +2
PEX1	c.1891delG	TGACTGT^630AAAgCTTTACGAG_E11I11_G
PEX1	c.1886_1887delGT	AGAGTT^628GACTgtAAAGCTTTAC
PEX1	c.1865_1866insGAGTGTGGA	AACTG^621GATGCgagtgtggaCCATGTGGAG
PEX1	c.1842delA	CAATC^613TGTAAaGAAGCATTTG
PEX1	c.1769T>G	Leu590Arg
PEX1	c.1727dupG	GCCTC^575CTGGGgACGCCCTTTG
PEX1	c.1716_1717delCA	GCACATC^571ACTcaCAGCCTCCTG
PEX1	c.1713_1716delTCAC	AGCAC^570ATCACtcacAGCCTCCTGG
PEX1	c.1706A>C	His569Pro
PEX1	c.1663T>C	Ser555Pro
PEX1	c.1508_1509dupTA	TCTGAGT^503ATAtaGTTCATTCTT
PEX1	c.1501_1502delCT	GGAATTT^500TCTctGAGTATAGTT
PEX1	c.1483+1G>A	IVS7 ds G-A +1
PEX1	c.1435_1439dupCCTTT	TGCTT^479CCTTTcctttGGTAATATCA
PEX1	c.1414C>T	Gln472Term
PEX1	c.1411C>T	Gln471Term
PEX1	c.1370dupT	CTAAA^456GACATtAAGTGAAGAA
PEX1	c.1340T>C	Leu447Ser
PEX1	c.130-2A>T	IVS1 as A-T -2
PEX1	c.1239+1G>T	IVS5 ds G-T +1
PEX1	c.1193delT	AACAAT^397GCCAtCAAATATACC
PEX1	c.1108dupA	AGATCAA^368AAAaAAAATTAGGT
PEX1	190 bp within ex. 12-13	
PEX1	190 bp nt. 2227-2416	
PEX1	171 bp nt. 1900-2070, cd.634-690	

PEX26	c.185G>A	Trp62Term
PEX26	c.256T>C	Cys86Arg
PEX26	c.265G>A	Gly89Arg
PEX26	c.315G>A	Trp105Term
PEX26	c.230+1G>A	IVS1 ds G-A +1
PEX26	c.73_79delGTGCGCG	CAGCGAG^24CCGgtgcgcgCGGTCCCGGC
PEX26	c.34dupC	TCTGCA^10GCCCcCCCTCAGGGG
PEX26	c.668_814del	gDNA
PEX26	c.426_548dup122insT	
